# *Rickettsia* and *Ehrlichia* of Veterinary and Public Health Importance in Ticks Collected from Birds in the Great Plains of the United States

**DOI:** 10.3390/pathogens14050461

**Published:** 2025-05-08

**Authors:** Tucker Taylor, Scott R. Loss, Bruce H. Noden

**Affiliations:** 1 Department of Entomology and Plant Pathology, Division of Agricultural Sciences and Natural Resources, Oklahoma State University, Stillwater, OK 74078, USA; tucker.taylor@okstate.edu; 2 Department of Natural Resource Ecology and Management, Division of Agricultural Sciences and Natural Resources, Oklahoma State University, Stillwater, OK 74078, USA; scott.loss@okstate.edu

**Keywords:** tick-borne pathogens, ticks, birds, landscape epidemiology, *Ehrlichia*, *Rickettsia*

## Abstract

As the incidence of tick-borne disease expands globally, comprehensive understanding of pathogen reservoir hosts is crucial to protect humans and wildlife. While many components are understood, there are gaps in our knowledge regarding the role of alternative, non-mammalian hosts such as birds. Within the United States, birds have been identified as reservoirs for *Borrelia* and *Rickettsia*; however, local studies rarely examine the potential of birds as reservoirs and transporters of *Ehrlichia*-infected ticks, unlike studies in Europe and South America. To address this research gap, we extracted and sequenced important microorganisms within 90 larval and nymphal ticks which were removed from passerine and near-passerine birds in the Great Plains region of the United States between May and October 2023. We found that 11% of birds hosted ticks infected with one or more *Rickettsia* or *Ehrlichia* species. Additionally, we collected a larval *Haemaphysalis leporispalustris* infected with *Ehrlichia chaffeensis* from a Northern Cardinal, the first North American songbird implicated in the *Ehrlichia* transmission cycle. Our research intertwines multiple bird and tick species in the North American pathogen system, highlighting the need for continued research focusing on birds as tick hosts and pathogen reservoirs in understudied parts of the United States.

## 1. Introduction

A tick-borne disease can occur when a reservoir host, a susceptible host, a competent arthropod, and an infectious microorganism all occur at the same time amid specific environmental and ecological conditions (i.e., ‘nidus of infection’ [1]). Although much is known concerning the relationships among vectors, hosts, and microorganisms [2], gaps in our knowledge regarding understudied alternative hosts remain [3]. The main reservoir hosts for most tick-borne bacterial pathogens in the United States are mammals, including white-tailed deer, canines, racoons, and rodent species [4,5]. Due to the importance of mammals, much research has focused on this host group [5,6,7]; however, birds can also be infested with pathogen-infected larval and nymphal stages of a variety of tick species [8,9,10,11] and additional research is needed to identify the ticks and tick-borne pathogens carried by birds. Among the studies evaluating avian hosts of tick-borne pathogens in the United States, most have focused on Lyme disease (*Borrelia burgdorferi*) [10,12,13,14] and most have been limited to the northern regions of the United States where the northern clade of *Ixodes scapularis* is the dominant vector [15]. In the southern and central United States where the highest incidence of Spotted Fever Group Rickettsiosis (SFGR) and Ehrlichiosis occurs, the primary tick species involved in pathogen transmission is the Lone star tick (*Amblyomma americanum*) [16,17,18]. Studies involving ticks collected from birds in the southern U.S., to date, have largely focused on the role of migratory birds in bringing infected ticks into this region [19,20,21,22,23]. Fewer studies have addressed the role birds play in contributing to the maintenance of common regional pathogens [5,24], despite a study in Oklahoma, USA, documenting high tick infestation rates of birds [25] as well as studies in Europe and South America that found birds can carry *Rickettsia* and *Ehrlichia*-infected ticks [26,27]. Thus, additional research in central and southern U.S., including the Great Plains region, is needed to determine the degree to which birds carry ticks infected with pathogens of concern to human health.

A confirmed benefit of sampling ticks directly from birds is that they can be used to gauge the likelihood of active pathogen infection in the host. Larval ticks can be a direct indicator of the presence of tick-borne pathogens in the blood of their hosts [28,29] because —with the exception of *Rickettsia* spp., which are transmitted transovarially [30,31]—larvae are free of certain bacterial pathogens before feeding on their first host [32]. To begin to assess the role of birds in contributing to the distribution of tick-borne bacterial species in the southern Great Plains, the aim of this descriptive study was focused on determining whether birds in this region carry ticks that are infected with pathogens of concern to human and animal health. We hypothesized that birds in Oklahoma, USA, are involved in the maintenance and spread of ticks infected with various bacteria, including *Rickettsia* and *Ehrlichia* species.

## 2. Materials and Methods

### 2.1. Site Selection

**Figure 1:**
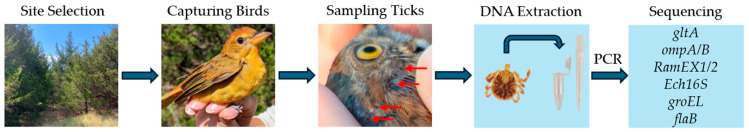
Overview of project materials and methods. Arrows imply next steps in the protocol.

In April 2022, we established nine sites across western and central Oklahoma in which we captured birds to sample them for ticks (Figure 1). These sites were selected as part of a larger study of 28 sites that focused on the effect of woody plant encroachment into grasslands, specifically by Eastern Redcedar (*Juniperus virginiana*), on tick abundance [33]. To select these sites, we identified publicly accessible areas (e.g., state parks/wildlife management areas; properties owned by Oklahoma State University [OSU]) using Google Earth, ESRI ArcGIS Pro 3.1.0 (ESRI, Redlands, CA, USA), and the Oklahoma Ecological Systems Mapping data layer (OESM), a 10 × 10 m resolution land cover layer that covers all of Oklahoma and includes specific designation of land cover types [34]. All details of site selection have been described [33,35]. Once candidate sites were identified, we worked with staff at parks, wildlife management areas, and properties owned by OSU to coordinate permission and access for research purposes. The nine sites used in the current study were at the Lake Carl Blackwell Family Recreation Area and OSU Range Research Station in central Oklahoma and Boiling Springs State Park, Fort Cobb State Park, American Horse Lake, and Canton Wildlife Management Area in western Oklahoma.

### 2.2. Capturing Birds

From mid-May to October of 2023, we performed mist-netting at the nine sites to collect ticks off live birds. As *A. americanum* is the dominant tick species in our study area [36], we attempted to sample at each site three times: once in late-May/early-June corresponding to this species’ peak nymphal season [36]; once in late July corresponding to its peak larval season; and once in late August/early-October, a period when this species is still highly active and abundant [37]. Due to weather constraints, we were unable to sample once at four of the sites which limited us to 23 total collection days. The order of sites sampled was randomly generated before each sampling season, although minor alterations to the sampling schedule occurred in some cases due to weather.

On each sampling day, we captured birds using six to eight mist nets (2.6 m in height, 12 m in length, 36 mm mesh, Avinet Inc., Dryden, NY, USA) that were opened between roughly sunrise and 10:30 AM, except when extreme heat or precipitation caused us to close nets early. The sites we used were previously open grasslands that, at the time of sampling, were experiencing varying levels of eastern redcedar encroachment. Mist nets in areas with light-to-moderate eastern redcedar cover were placed along the edges of individual trees or tree clusters; nets in areas with advanced encroachment with extensive tree cover and closed canopies were placed along corridors within eastern redcedar woodlands. Bird capture and handling was permitted under a U.S. Geological Survey federal bird banding permit (#23929), an Oklahoma Department of Wildlife Conservation permit (#W2304), and approved by the Institutional Animal Care and Use Committee at Oklahoma State University (permit #23-09-STW). Since little is known about which bird species carry ticks in areas experiencing eastern redcedar encroachment, any passerine or near-passerine (in our case, one woodpecker and one cuckoo species) caught in the net was examined for ticks.

### 2.3. Sampling Ticks

For all captured birds, we used a protocol previously developed in the same region [38] to thoroughly check all body parts (head, neck, underwing, flanks, legs) for the presence of ticks by blowing or pushing feathers aside to check the skin of the bird (Figure 1). To prevent undue stress or injury, we released one bird without performing a tick check and incidentally captured three birds for which we did not have permits to handle (two hummingbirds and one owl) that we immediately released. Ticks were carefully removed from birds in the hand using needle-tipped forceps and placed immediately into a 1.5 mL tube containing 70% ethanol. We recorded the number and species of ticks associated with each bird and date of sampling on a data sheet. Ticks were identified to species in the lab within two weeks of sampling using pictorial keys [39,40,41,42]. Due to the focus of this manuscript on the microorganisms detected, the comprehensive tick abundance and species composition data along with the infestation rates have been reported in another manuscript [33].

### 2.4. DNA Extraction

Individual nymphal ticks were placed in a 1.5 mL tube with 15 grains of zirconium powder and 40 uL PBS as emulsifier, then macerated by a heat-sealed pipette tip as a pestle. Macerated individual nymphal ticks were column extracted following the manufacturer’s protocol (Qiagen DNA Blood and Tissue Kit [Qiagen, Hilden, Germany]) to remove blood byproducts, which can interfere with the amplification process [43]. Individual larval ticks were macerated by a heat-sealed pipette tip in 1.5 mL tubes with 5 uL of PBS after which 15 uL of QuickExtract DNA Extraction solution (LGC Genomics, Alexandria, MN, USA) was added and a protocol was followed that was similar to Goethert et al. [44].

### 2.5. Screening and Sequencing

Due to the high incidence of Spotted Fever Group Rickettsia and Ehrlichiosis in the region [16], individual ticks were screened using previously published PCR protocols (Supplemental Table S1) targeting the *Rickettsia gltA* gene (CS78F/CS323R) [45], the *Rickettsia ompA* gene (190.70p/190-701) [46,47,48], and the *Ehrlichia 16S rRNA* gene [49,50] while we used primers targeting the tick-specific *16S rRNA* gene (TQ16S+1F/TQ16S-2R) to confirm larval tick identification [51,52]. If enough template was available, we confirmed the *Rickettsia* species by using primers targeting the *ompB* gene (120-2788/120-3599) [53], the *Ehrlichia* species by using nested primers targeting the *groEL* gene [54,55], and *I. scapularis* larvae and nymphs using a nested PCR assay targeting the flagellin (*flaB*) gene of *Borrelia burgdorferi* [56,57]. To limit DNA contamination, all tick DNA extractions were conducted using site-specific reagents in a different laboratory than where PCR assays were run. Positive controls consisted of *R. rickettsii* DNA provided by Dr. William Nicholson (Rickettsial Zoonoses Branch, Centers for Disease Control and Prevention), *E. chaffeensis* MO strain DNA provided by Dr. Susan Little (OSU School of Veterinary Science), and *Borrelia burgdorferi* strain B31 DNA acquired from American Type Culture Collection (Manassas, VA, USA), while the negative control consisted of reagents with no template added.

All amplicon-positive samples were bidirectionally sequenced at the Oklahoma State University Core Facility to confirm positivity and identify bacterial species. We verified each resulting sequence using BioEdit 7.7 (Ibis Therapeutics, Carlsbad, CA, USA), aligned to create consensus sequences using Clustal Omega (EMBL-EBI, Cambridgeshire, UK). We compared resulting consensus sequences with GenBank submissions using default conditions on NCBI BLAST (highly similar sequences [megablast]) where the highest % sequence identity [99–100%] was used to determine species identity.

## 3. Results

### 3.1. Overall Results

Of 140 birds sampled, 25.7% (36/140) were infested with at least one tick. No adult ticks were found on any captured birds as only larvae and nymphs were collected from birds. Of 90 ticks screened, 22.2% (19/90) contained DNA for at least one microorganism and 2.2% (1/90) were co-infected, resulting in 10.7% (15/140) of all birds sampled hosting at least one tick containing DNA from one or more microorganisms (Table 1). In total, 7.9% (11/140) of the birds sampled hosted ticks containing DNA from *R. amblyommatis* and *Ehrlichia*, species that have been demonstrated to produce infections in canines and humans. Of the screened ticks, 17.8% (16/90) were infected with a *Rickettsia* species and 4.4% (4/90) were infected with an *Ehrlichia* species.

### 3.2. Prevalence of Rickettsia by Tick Species

The infection rate for all *A. americanum* nymphs tested was 18.0% (9/50). In total, 22.5% (9/40) of the amplicon-positive *A. americanum* nymphs from three *Cardinalis cardinalis* (Northern Cardinal), one *Passerina ciris* (Painted Bunting), one *Passerina cyanea* (Indigo Bunting), and one *Piranga rubra* (Summer Tanager) as well as 100% (1/1) of *A. americanum* larvae from a Northern Cardinal were 100% identical to *Rickettsia amblyommatis* (*gltA* and *ompA*: GenBank accession no. CP012420) (Table 2). One amplicon-positive larval *Amblyomma maculatum* from a Painted Bunting produced a sequence that was 100% identical with ‘*Candidatus* Rickettsia andeanae’ (*gltA* and *ompA*: GenBank accession no. OR885255 and OR885258). Three amplicon-positive *I. scapularis* larvae from two *Thryothorus ludovicianus* (Carolina Wren) were 100% identical to *Rickettsia buchneri* (*ompA*: GenBank accession no. CP113531).

Two amplicon-positive *I. scapularis* nymphs were collected, one from a Carolina Wren that was 100% identical to *R. buchneri* (*ompA*: GenBank accession no. CP113531) and the other from a Painted Bunting that was 100% identical to an unknown *Rickettsia* spp. [58,59] (*ompA*: GenBank accession no. KX002001), and also 97% identical to *R. rhiphicephali* (*ompB*: GenBank accession no. CP013133). Four sequences involving consensus sequences for each *Rickettsia* species detected in ticks removed from birds were deposited in GenBank (accession numbers PV296324-PV296327)) (Table 2).

We documented ticks infected with *R. amblyommatis* throughout the season, beginning in May until September, while all *Rickettsia*-infected *I. scapularis* were collected at four different sites only in June (Table 2). Focusing on the 15 birds with at least one tick with microorganismal DNA, ten (71.4%) of the birds also were infested with ticks that were not positive for microorganisms, including the Painted Bunting that produced a co-infected tick as well as an infected *A. maculatum* nymph.

### 3.3. Prevalence of Ehrlichia by Tick Species

In total, 4.0% (2/50) of the *A. americanum* nymphs, one each from a Northern Cardinal and a Painted Bunting, were 100% identical to the *Ehrlichia chaffeensis* Arkansas strain (*Ech16S*: GenBank accession no. NR074500), one of which was co-infected with *R. amblyommatis* and *E. chaffeensis* (from a Painted Bunting) (Table 2). One amplicon-positive *A. americanum* nymph from a Northern Cardinal produced a sequence that was 99% identical to *Ehrlichia ewingii* Stillwater strain (*Ech16S*: Genbank accession no. M73227).

One (3.3%) amplicon-positive *H. leporispalustris* larvae from a Northern Cardinal was 100% identical for *E. chaffeensis* (*Ech16S*: GenBank accession no. CP000236). No ticks collected from birds were amplicon-positive for *groEL*. All *Ehrlichia* sequences identified from ticks removed from birds were deposited in GenBank (accession numbers PV274861-PV274864) (Table 2).

We documented ticks infected with *Ehrlichia* throughout the season, beginning in May until October (Table 2). The Northern Cardinal that was infested with the *E. chaffeensis*-infected *H. leporispalustris* larvae was also infested with three other larvae which did not test positive for any microorganism. This infected *H. leporispalustris* was collected in October which was later in the season than the other *Ehrlichia* samples on *A. americanum*.

### 3.4. Borrelia and Tick-Specific Assays

No *I. scapularis* larvae or nymphs collected from birds were amplicon-positive for *Borrelia* spp. (*flaB*). The larvae identifications were confirmed molecularly using *16S rRNA* primers to species.

## 4. Discussion

This study provides evidence that up to 8% of birds that we sampled in the southern Great Plains region of the U.S. are infested with ticks contained several microorganisms that may cause disease in humans and domestic animals. Addressing the hypothesis for the study, several *Rickettsia* species identified (‘*Candidatus* R. andeanae’ and *R. buchneri*) are not associated with pathogenesis in animals, while *R. amblyommatis* has produced SFG Rickettsia infections in companion animals and possibly in humans [17,60,61]. Most of the bacterial species detected are transovarially transmitted between adult females and their progeny (*R. amblyommatis*, ‘*Candidatus* R. andeanae’, *R. buchneri*), but not *Ehrlichia chaffeensis* [62]. This study is the first to identify *E. chaffeensis* in a larval rabbit tick, *H. leporispalustris*, from a ground-foraging Northern Cardinal in the United States.

North American studies have reported that many bird species have the potential to be reservoirs for *B. burgdorferi* [5,12,14,63,64,65], the tick-borne pathogen that causes Lyme disease in the U.S. [66]. However, few North American studies have evaluated the prevalence of *Ehrlichia* species in bird blood or larvae which have fed on birds, testing which has been recently conducted in Europe and South America [28,67,68]. In North America, *Ehrlichia* species are primarily considered to be transmitted by *A. americanum* with white-tailed deer as the primary reservoir [69]. The detection of *E. chaffeensis* in a larval blood-fed *H. leporispalustris*, a species not considered competent for this pathogen, likely indicates that the bird from which the tick was extracted was infected and DNA from the bird’s blood was present in the larval tick [28]. Co-feeding from another tick on the same bird does not seem likely due to the lack of detectable bacterial DNA in the three other larvae on the same bird and the absence of infected *A. americanum* on the bird. The presence of *E. chaffeensis* in a singular tick may be due to the other larvae not feeding long enough to detect bacterial DNA by PCR in the infected blood. The *E. chaffeensis* sequence obtained (PV274864) was identical to the Arkansas strain genotype which is expected given the sequences were obtained in the south-central region of the U.S. *Ehrlichia chaffeensis* has previously been detected in the blood of a falcon species, specifically a Crested caracara (*Caracara cheriway*) from Florida [24], but this appears to be the first detection of this pathogen in a North American resident songbird (Northern Cardinal). While two *E. chaffeensis*-infected *A. americanum* nymphs and one *E. ewingii*-infected *A. americanum* nymph were collected from two Northern Cardinals and one Painted Bunting, the ‘catholic’ feeding habits of this tick species make it likely that the larvae had fed on a known *Ehrlichia* reservoir, likely a white-tailed deer, prior to the nymphs feeding on birds. To date, white-tailed deer are considered to be the main reservoir involved in the transmission of *E. chaffeensis* to *A. americanum* ticks [70]. Similar to our results, a recent study in southern Texas detected *E. chaffeensis* and *E. ewingii* in nymphal ticks removed from birds that had probably fed on deer as larvae [71]. If birds are also *E. chaffeensis* reservoirs, they could contribute to both local and larger-scale movements of infected ticks that influence spatial and temporal distributions of pathogens, as well as large-scale patterns of disease risk that have important public health implications.

Unlike the *Ehrlichia* species, most of the *Rickettsia* species detected in ticks collected from birds in Oklahoma contain species known to be transovarially transmitted. Thus, the detection of *R. amblyommatis* in *A. americanum* larvae, ‘*Candidatus* Rickettsia andeanae’ in *A. maculatum* larvae, and *R. buchneri* in *I. scapularis* larvae is expected, irrespective of the host [72,73]. Similar *Rickettsia*-positive results have been reported from both birds and ticks on birds collected in North America [22,74] and Europe [26]. We documented that an *I. scapularis* nymph collected from a Painted Bunting was infected with an unknown *Rickettsia* spp., which has been reported in *I. scapularis* in Florida [57] and East Texas [58] but remains to be characterized at the organismal level. The detection of a novel *Rickettsia* spp. in a tick parasitizing a bird indicates that there is a general need for additional research into the role of birds as potential reservoir hosts. While considered endosymbionts, the potential for *R. amblyommatis* to be involved with the increased SFG Rickettsia incidence in the regions of the U.S. where *A. americanum* predominates [17] indicates that local birds may be more involved in the maintenance and dispersion of infected ticks that previously thought.

While small in scale, this study provided results that indicate a need for more focused follow-up research. First, the lack of *B. burgdorferi* infection in any of the *I. scapularis* was expected. Unlike northern areas of the U.S., Oklahoma resides in the region of the U.S. where *I. scapularis* feed on reptiles and possibly birds (which is covered in more detail in our parallel paper [33]) [75]. What was unexpected, however, was the collection of *R. buchneri*-infected larval and nymphal *I. scapularis* on two different bird species from four geographically different locations all within days of each other at the end of June. Although possibly just a coincidence, it also may be that this period is the primary time when larval *I. scapularis* seek out avian hosts instead of reptiles [75]. Second, the seasonality of ticks associated with microbial infections was unique. The ticks infected with *Rickettsia* and *Ehrlichia* species were collected during the period when larvae and nymphs are most active in the region [36]. While the phenology of *H. leporispalustris* is not well characterized in Oklahoma, the presence of an *Ehrlichia*-infected larvae on a bird in October matches the August–October time range during which this species has been reported on birds in other regional studies [76,77].

We recognize that some limitations occurred in the completion of this study. As a pilot study, we were relatively limited in the number of sampling days and net hours. This provided relatively few comparison points for bird and tick species captured only on one day during the study duration, but our wide geographic sampling area across different seasons still illuminated unique host–tick pairings. Additionally, we did not pair results of infected ticks with blood samples taken directly from the birds themselves. The screening for DNA from blood-fed larvae, however, is a cheap and effective sentinel system for identifying potential avian reservoir hosts for specific pathogens, and this method is used in Europe and South America [28,67,68]. Although we were able to screen the samples with several PCR primers focused on *Ehrlichia*, the limited amount of template provided by our larval extraction methods on individual ticks meant that we did not have enough template to characterize using other primers. Although we are assured that the sequences were identical to known *Ehrlichia* species, additional studies are needed to further characterize the *Ehrlichia* species detected.

## 5. Conclusions

This study in the Great Plains region of the United States demonstrates that a high proportion of birds may either be actual reservoirs or effective distributors of pathogen-infected ticks. We continue to find that approximately 25% of birds in this region, in both urban and rural settings, are infested with ticks [25,33]. In addition, our pathogen assessment in the current paper demonstrated that about 8% of the birds captured were infested with ticks that were infected with microorganisms which may produce infections in humans or companion animals. Hypothetically, our results would suggest that out of every 400 birds in the southern Great Plains, about 100 (25%) would be infested with ticks. In total, 32 (8% of 400) of those hypothetical tick-infested birds (or 32% (32/100) of the birds with ticks) could drop off a tick with a bacterial pathogen that could infect humans and companion animals. This prevalence of potential pathogen-infected ticks on birds could have important ramifications for the epidemiology of tick-borne pathogens in the region. Researchers should consider incorporating bird dynamics into the determination of pathogen and tick hotspots, just as white-tailed deer and white-footed mice population density metrics are used today [78,79]. For instance, future studies could assess whether integration of bird abundance or occurrence data, including bird species documented to carry a large number of infected ticks, improves predictions about the risk of encountering infected ticks or the prevalence of tick-borne diseases in the Great Plains. Ultimately, the role birds play in the maintenance of tick-borne pathogens presents an important direction for avian research useful for public health efforts focused on reducing the incidence of vector-borne disease.

## Figures and Tables

**Table 1:** Summary of birds infested with ticks containing *Rickettsia* and *Ehrlichia* species that were collected from birds across nine sites in central and western Oklahoma, from May to October 2023.

Bird Species	Total Birds Captured/w Ticks	Total Birds w Micro-Organisms in Ticks	Tick Species	Tick Lifestage	Total Ticks Tested	*Rickettsia* (%)	*Ehrlichia* (%)
**Brown Thrasher** (*Toxostoma rufum*)	1/1	0	*Aa*	N	5	0	0
**Carolina Wren** (*Thryothorus ludovicianus*)	5/3	3	*Aa*	N	4	0	0
			*Is*	L	3	3 (100.0)	0
				N	1	1 (100.0)	0
**Common Grackle** (*Quiscalus quiscula*)	6/1	0	*Aa*	L	1	0	0
**Indigo Bunting** (*Passerina cyanea*)	4/2	1	*Aa*	N	2	1 (50.0)	0
**Northern Cardinal** (*Cardinalis cardinalis*)	53/26	8	*Aa*	L	1	1 (100.0)	0
				N	35	6 (17.1)	2 (5.7)
			*Hl*	L	30	0	1 (3.3)
				N	2	0	0
**Painted Bunting** (*Passerina ciris*)	11/2	2	*Aa*	N	2	1 (50.0)	1 (50)
			*Am*	L	1	1 (100.0)	0
			*Is*	N	1	1 (100.0)	0
**Summer Tanager** (*Piranga rubra*)	2/1	1	*Aa*	N	1	1 (100.0)	0
**Swainson’s Thrush** (*Catharus ustulatus*)	1/0	0	*Aa*	N	1	0	0
**Other bird species** without ticks **	57/0	0					
**Total**	140/36	15			90	16 (17.8)	4 (4.4)

Aa = *Amblyomma americanum*, Am = *Amblyomma maculatum*, Hl = *Haemaphysalis leporispalustris*, Is = *Ixodes scapularis*. ** For a detailed list of all birds sampled, including those without ticks, see [33].

**Table 2:** Details of birds infested with ticks infected with *Rickettsia* and/or *Ehrlichia* species across nine sites in central and western Oklahoma, from May to October 2023.

Bird Species	Collection Location *	Collection Date	Tick Species ^	Tick Lifestage ^X^	# Other Ticks Same Bird	*Rickettsia* sp. ^‡^ (NCBI #)	*Ehrlichia* sp. ^‡^ (NCBI #)
**Carolina Wren** **1**	CB	22 June 2023	*Is*	L	2 uninfected	*R. buchneri* (PV296324)	
**2**	FW	26 June 2023	*Is*	L	1 uninfected	*R. buchneri*	
**3**	FE	27 June 2023	*Is*	L	1 uninfected	*R. buchneri*	
				N		*R. buchneri*	
**Indigo Bunting** **1**	FW	1 September 2023	*Aa*	N	1 uninfected	*R. ambly.* (PV296326)	
**Northern Cardinal 1**	BS	7 September 2023	*Aa*	L	0	*R. ambly.*	
**2**	FE	30 May 2023	*Aa*	N	1 uninfected		*E, chaff.* (PV274862)
**3**	CB	22 June 2023	*Aa*	N	4 uninfected		*E. ewingii* (PV274861)
**4**	RR	21 May 2023	*Aa*	N	2 uninfected	*R. ambly.*	
**5**	RR	21 May 2023	*Aa*	N	1 uninfected	*R. ambly.*	
**6**	CA	14 July 2023	*Aa*	N	0	*R. ambly.*	
				N		*R. ambly.*	
**7**	FE	2 September 2023	*Aa*	N	0	*R. ambly.*	
				N		*R. ambly.*	
**8**	CA	7 October 2023	*Hl*	L	3 uninfected		*E. chaff.*
**Painted Bunting** **1**	CA	14 July 2023	*Aa*	N	1 uninfected	*R. ambly.*	*E. chaff.*
			*Am*	L		‘*Can.*’ R. andeanae (PV296325)	
**2**	RR	23 June 2023	*Is*	N	0	Unknown Rickettsia (PV296327)	
**Summer Tanager 1**	CB	23 May 2023	*Aa*	N	0	*R. ambly.*	

* CB—Lake Carl Blackwell, FW—Ft. Cobb West, FE—Ft. Cobb East, BS—Boiling Spring SP, CA—Canton WMA, RR—OSU Research Range. ^ Is—*I. scapularis*, Aa—*A. americanum*, Hl—*H. leporispalestris*, Am—*A. maculatum*. ^X^ L—larvae, N—nymph. ^‡^ R. ambly.—*R. amblyommatis*, ‘*Can*.’ R andeanae—‘*Candidatus*’ R. andeanae, *E. chaff*.—*E. chaffeensis.*

## Data Availability

Data for this publication are fully presented in this manuscript. Additional information can be found in [33].

## Supplementary Materials

The following supporting information can be downloaded at: https://www.mdpi.com/article/10.3390/pathogens14050461/s1. Table S1. Protocols used for testing ticks for selected microbial agents and molecular tick confirmation.
