# Supplementary material for: Rickettsia and Ehrlichia of Veterinary and Public Health Importance in Ticks Collected from Birds in the Great Plains of the United States
Source: Pathogens. 2025 May 8;14(5):461. doi: 10.3390/pathogens14050461 (PMC12114804; doi:10.3390/pathogens14050461)
Supplement: Supplementary file 1 [file pathogens-14-00461-s001.zip › pathogens-3582377-supplementary.pdf]

Supplemental Table 1. Protocols used for testing ticks for selected microbial agents and molecular tick confirmation

Organism	Gene Target	Primers (5'-3')	Annealing temp	Reference
**Spotted fever group *Rickettsia***	Citrate synthase (*gltA*)	CS-78: GCAAGTATCGGTGAGGATGTAAT	48 °C	[45]
		CS-323: GCTTCCTTAAAATTCAATAAATCAGGAT		
	Outer membrane protein A (*ompA*)	190.70p: ATGGCGAATATTTCTCCAAAA	46 °C	[46-48]
		190-701: GTTCCGTTAATGGCAGCATCT		
	Outer membrane protein B (*ompB*)	M59F: CCGCAGGGTTGGTAACTGC	50 °C	[53]
		807R: CCTTTTAGATTACCGCCTAA		
Ehrlichia sp.	*Ech16S*	SYBR-F: AACACATGCAAGTCGAACGG	60 °C	[49,50]
		SYBR-R: CCCCCGCAGGGATTATACA		
Ehrlichia and Anaplasma	*groEL* (Outer)	607F: GAA GAT GC(A/T) GT(A/T) GG(A/T) TGT AC(G/T) GC	57 °C	[54,55]
		1294R: AG(A/C) GCT TC(A/T) CCT TC(A/T) AC(A/G) TC(C/T) TC		
	groEL (Inner)	677F: ATT ACT CAG AGT GCT TCT CA(A/G)	57 °C	
		1121R: TGC ATA CC(A/G) TCA GT(C/T) TTT TCA AC		
***Borrelia* spp.**	*flaB* (Outer)	LLACATATTCAGATGCAGACAGAGGT	55 °C	[56,57]
		RL: GCAATCATAGCCATTGCAGATTGT		
	flaB (Inner)	S: AACAGCTGAAGAGCTTGGAATG	55 °C	
		RS: CTTTGATCACTTATCATTCTAATAGC		
**Tick Species**	16S rRNA	F: CGTAAAGGGCACGTAGGTGGACTA	55 °C	[51,52]
		R: CACCTCAGTGTCAGTATCGAACCA		
